# Immune-Boosting Potentiating Properties of *Brassica nigra* Hydroalcoholic Extract in Cyclophosphamide-Induced Immunosuppression in Rats

**DOI:** 10.3390/foods12193652

**Published:** 2023-10-03

**Authors:** Hassan Barakat, Raghad I. Alkhurayji, Thamer Aljutaily

**Affiliations:** 1 Department of Food Science and Human Nutrition, College of Agriculture and Veterinary Medicine, Qassim University, Buraydah 51452, Saudi Arabia; 421200388@qu.edu.sa (R.I.A.); thamer.aljutaily@qu.edu.sa (T.A.); 2 Food Technology Department, Faculty of Agriculture, Benha University, Moshtohor 13736, Qaliuobia, Egypt

**Keywords:** *Brassica nigra*, polyphenols, immune boosting, oxidative stress, innate immunity

## Abstract

The antioxidative and immune-boosting properties of the hydroalcoholic extract of *Brassica nigra* sprouts in cyclophosphamide-induced immunosuppression in rats were investigated in this study. *B. nigra* sprouts were prepared in the lab to monitor the bio-changes in bioactive compounds during the sprouting period up to 7 days at 17 ± 1 °C and 90% relative humidity. The total phenolic content (TPC), antioxidant activity (AOA), total flavonoids (TFs), total flavonols (TFLs), and total carotenoids (TCs) were evaluated. Consequently, the identification and quantification of phenolic acids, their derivatives, and flavonoids were carried out using HPLC. Subsequently, the selected BN sprout (6-day-old sprout) was biologically examined, and oxidative stress biomarkers, hematological parameters, immunoglobulins (Igs), and pro-inflammatory and anti-inflammatory cytokines were investigated. An increase in TPC, AOA, TFs, TFLs, and TCs was observed by increasing the sprouting time. The HPLC analysis indicated that the *B. nigra* seeds contained 10 phenolic acids and 4 flavonoids, predominantly syringic acid and quercetin, respectively. After 3 days, the number of phenolic acids increased to 16, predominantly syringic acid, and the number of flavonoids increased to 7, predominantly quercetin. On the 6th day, 13 phenolic acids were estimated, with the highest being benzoic acid, and 6 flavonoids were estimated, with the highest being quercetin. The greatest rise in phenols was seen on the sixth day of sprouting. These included caffeic acid, chlorogenic acid, cinnamic acid, ferulic acid, coumaric acid, benzoic acid, and rosmarinic acid. Flavonoids such as kaempferol and myricetin increased. The sprouts on day 6 were recorded as having the highest bioactive compounds and AOA content. The selected *B. nigra* sprouts were examined for antioxidative and immunomodulatory properties in a rat model. Dosing 250 and 500 mg kg^−1^, the rats exhibited significant improvements in terms of antioxidative stress and the number of white blood cells (WBCs), lymphocytes, and neutrophils in the blood, indicating stimulation of the immune response in a dose-dependent manner. In addition, the production of immune proteins, such as IgG, IgM, and IgA, was enhanced in the blood. Moreover, the 500 mg kg^−1^ concentration of BN extract stimulated cytokine production in a stronger manner than the 250 mg kg^−1^ concentration, indicating that the extract significantly increased immune activity. In conclusion, the results indicate that mustard seed extracts have immunosuppressive properties against cyclophosphamide-induced immunosuppression in rats.

## 1. Introduction

Plant-based food sources significantly improve immune system performance and disease resistance, and they have significant preventative and therapeutic advantages. The WHO estimates that up to 80% of people in underdeveloped nations rely on locally available plant resources because traditional medicines are out of reach and prohibitively expensive compared with natural plants [1]. In this regard, Brassica is one of the most popular food plants in the world due to its useful applications [2]. The Brassica family’s phytochemicals impart functional qualities, with distinctive and numerous outstanding and advantageous biological activities provided by secondary metabolites [3]. Brassica is one of the main food sources of future food systems due to its high nutritional value, affordability, and widespread distribution and accessibility [4]. Relevant research supports Brassica vegetable production and its use as a staple food [5,6]. Phytochemicals and biological compounds, including polyphenols, phenolic acids, flavonoids, carotenoids (zeaxanthin, lutein, and β-carotene), alkaloids, phytosterols, chlorophyll, glucosinolates (isothiocyanates and argentine), terpenoids, and glucosides, are found in Brassica plants [7]. In addition, phytosterols, phytoalexins, terpenes, and tocopherols are also reported in Brassica plants [3,8]. Furthermore, they may boost immunity and bone health, exhibit anti-inflammatory and anti-cancer benefits, and protect against disease-related oxidative damage [6].

Mustard seed (*Brassica nigra*) is a Brassica plant; despite it being an excellent food source that is rich in proteins, vitamins, minerals, and other crucial nutrients for maintaining body health [9,10], it still needs more research attention, especially regarding its sprouts’ immunomodulatory properties, which have not yet been studied. However, *Brassica* plants may possess some therapeutic activities, as shown in an exemplary review of *Brassica juncea* [11]. The researchers found evidence of its potential antioxidation, anti-inflammatory, bacteriostatic, and antiviral properties. It is potentially useful in the fight against cancer, obesity, depression, diabetes, and cataracts. [11]. Interestingly, *B. nigra* seeds have anti-diabetic characteristics [12], as they have been shown to increase the activity of glucose-producing enzymes and reduce those of glycolytic enzymes in the liver and renal tissues of rats, as well as cure several metabolic illnesses [13]. Alam et al. [14] stated that the ability of *B. nigra* to reduce inflammation depends on the presence of sinigrin, which can prevent the activation of p65, NOD-like receptors (NLRP-3), and mitogen-activated protein kinase (MAPK) [15]. Also, *B. nigra* extract reduces free radicals and has cellular antioxidative activity, increasing the superoxide dismutase (SOD) level [16]. In addition, *B. nigra* extract has been shown to have membrane-stabilizing properties. For example, terpenoids, found in both the seeds and leaves of *B. nigra*, are another reason for its anti-inflammatory effect [17]. Furthermore, Rajamurugan et al. [18] stated that *B. nigra* extract lacks inherent toxicity and exhibits nephroprotective effects against D-GalN-induced toxicity in rats. Similarly, Al-Qady and Shaban [19] confirmed the protective effect of *B. nigra* seed extract against the physiological and histological effects of captopril on the kidney. Moreover, in a recent study, the anti-cancer efficacy of *B. nigra* seeds against human lung cancer cell lines was confirmed [9].

Young greens and sprouted seeds sometimes have more nutrients than mature or ripe seeds or plants. Sprouts are special functional foods with much potential to sustainably improve the world’s food systems and human health [20,21]. Thus, plant-sprout-based meals are becoming more popular as functional foods, attracting the attention of researchers and practitioners worldwide [22]. While previous studies have examined the nutritional value of sprouted seeds and sprouts of various plants by examining their primary elements and derivatives and by evaluating their nutritional properties and roles in boosting immunity and human health, *B. nigra* seeds have rarely been studied despite their nutritional significance. For instance, Western Europe has increased its intake of sprouts due to their nutritious value [23]. Indeed, sprouts may be described as a predigestion food since they promote enzyme activity that breaks down proteins, carbohydrates, and fatty acids. This enhances glucose conversion into monosaccharides, oligosaccharides, free fatty acids, free amino acids, and lower-level peptides, which helps the body in growth and development [23]. Moreover, substances with possible health-preserving effects and phytochemical qualities, like glucosinolates and natural antioxidants, can also be discovered during sprouting [24,25].

Because of the factors mentioned above, a crucial question may be answered by investigating the immune-boosting potential of *B. nigra* sprouts. The answer to this query will help explain how *B. nigra* sprouts can strengthen the immune system. Obviously, consuming antioxidant-rich foods increases life expectancy, protects the body from illness and inflammation, and promotes an appealing and high-compliance approach. This study is significant because it will improve our knowledge of the nutritional and immunostimulant benefits of *B. nigra* sprouts and help develop a novel, efficient, and secure nutritional strategy for preventing infections and illnesses linked to the immune system. 

Therefore, the aim of the current research was to study the immune-boosting potentiating properties of the hydroalcoholic extract of *B. nigra* sprouts in cyclophosphamide-induced immunosuppression in experimental rats. To achieve this, *B. nigra* seeds were sprouted under laboratory conditions, and bioactive compounds were monitored during sprouting. Later, the total phenolic content (TPC), antioxidant activity (AOA), total flavonoids (TFs), total flavonols (TFLs), total carotenoids (TCs), and individual phenolic acids and flavonoids were quantified using HPLC to select the sprout richest in bioactive components. Subsequently, a hydroalcoholic extract of *B. nigra* was prepared and biologically assessed to investigate the oxidative stress biomarkers, hematological parameters, immunoglobulins (Igs), and pro- and anti-inflammatory cytokines in an immunosuppressed rat model.

## 2. Materials and Methods

### 2.1. Raw Mustard Seeds

Mustard seeds (*B. nigra* L.) were purchased online from True Leaf Market (www.trueleafmarket.com). Two kilograms of pure seeds was obtained and stored in a dry and cool place until use.

### 2.2. Preparation of Sprouts

The raw mustard seeds were sieved using stainless steel sieves to remove foreign materials and dust, and then they were processed in a laboratory mill to make a finer powder for analysis. Seeds were also sprouted in 50 g batches after being superficially sanitized via soaking in a 2% sodium hypochlorite solution for 2 min. The seeds were then rinsed three times with sterile distilled water (sd.H_2_O). In 32 × 22 × 8 cm plastic pans, the seeds were evenly dispersed between two layers of moist filter paper and inserted into a seed spreader. According to Barakat et al. [26], the sprouting process was carried out in a temperature-controlled seed germination machine with an atomizer (EasyGreen, Model: EGL 50, Canada) at 17 ± 1 °C and 90% relative humidity. The *B. nigra* seeds were sprayed daily with 5 mL H_2_O/dish for the first three days. For a phytochemical analysis, appropriate samples were collected daily up to 7 days after the start of the sprouting process. *B. nigra* sprouts were immediately frozen overnight at −18 ± 1 °C and lyophilized (CHRIST, Alpha 1–2 LD plus, Osterode, Germany) for 96 h at −48 °C and 0.032 mbar. The freeze-dried sprouts were ground in a mini-mill (Snijders Scientific Tilburg; Model: 8010E, the Netherlands), sieved (60-mesh sieve) to prepare a homogeneous powder, and then kept under cooling at 4 ± 1 °C in dark packages until further phytochemical and HPLC analyses. Additionally, to perform the biological evaluation of the *B. nigra* sprouts in vivo, 2 kg of *B. nigra* was sprouted separately under the same conditions for 6 days, gradually dried following a 24 h drying program [26], ground, sieved, and extracted.

### 2.3. Preparation of Extracts for Phytochemicals Analysis

An appropriate weight of freeze-dried *B. nigra* sprout was extracted with 70% methanol three times to determine TPC, AOA, TFs, and TFLs. To determine the TCs in *B. nigra*, appropriate samples were extracted three times with 85% acetone. For clarification, all extracts were centrifuged at 5000× *g* for 5 min and then stored at −18 ± 1 °C until the procedure analysis was carried out.

### 2.4. Determination of TPC, TCs, TFs, and TFLs in B. nigra Seeds and Their Sprouts

The TPCs in the *B. nigra* seeds and their sprouts were determined using the Folin–Ciocalteu protocol [27]. The absorbance was then measured at 765 nm using a microplate reader (BioTek, Winooski, VT, USA). A standard curve using a gallic acid (GA) solution was prepared to compare the measurements. The TPC content is expressed as milligrams of gallic acid equivalents (GAE) per g (mg of GAE g^−1^ dw). To determine TCs, one g of the freeze-dried sample was repeatedly extracted with a mixture of acetone and petroleum ether (1:1, *v/v*), according to Yuan et al. [28]. The upper phase was collected, washed several times with water, and combined with crude extracts. The petroleum ether was then added to the solution to obtain a known volume. The TC content was spectrophotometrically determined at 450 nm and is expressed as mg g^−1^ dw. The TF content in the *B. nigra* seeds and their sprouts was determined as Mohdaly et al. [29] described. The TFL concentrations in the *B. nigra* seeds and their sprouts were determined by reacting methanolic extract aliquots with sodium acetate (5%). AlCl_3_ (2%) was added after 5 min, and the optical density (OD) was measured after 150 min at 440 nm, as described by Kumaran and Karunakaran [30]. The TF and TFL concentrations are reported in milligrams of quercetin equivalents (mg QE) per gram of dry weight (mg QE g^−1^ dw).

### 2.5. Determination of Antioxidant Capacity 

According to Khalifa et al., radical scavenging activity was measured spectrophotometrically based on the bleaching of a DPPH radical solution [31]. The absorbance was then measured at 517 nm using a microplate reader (BioTek, Winooski, VT, USA). The antiradical activity is expressed as micromoles of Trolox equivalents (TE) per gram (µmol TE g^−1^ dw). The radical scavenging activity (RSA) of the *B. nigra* seeds and their sprouts against a stable ABTS (2,2′-azino-bis(3-ethylbenzothiazoline-6-sulphonic acid)) radical cation was measured using the adapted method of Lu et al. [32]. A Trolox calibration curve was then plotted as a function of the percentage of ABTS radical cation scavenging activity. The final results are expressed as micromoles of Trolox equivalents (TE) per gram (µmol TE g^−1^ dw).

### 2.6. Quantification of Phenolic Compounds in B. nigra Seeds and Their Sprouts Using HPLC-DAD

The phenolic compounds in the *B. nigra* seeds and their sprouts were determined using the HPLC system HP1100 (Agilent Technologies, Palo Alto, CA, USA) equipped with an autosampler, quaternary pump, and diode-array detector (DAD) (Hewlett Packard 1050), using a column (Altima C18, 5 × 150 mm, 4.6 mm ID) and a guard column (Altima C18, 5 mm) (Alltech) according to Kim et al. [33]. The solvent system contained a gradient of 95% (A) 2.5% acetic acid in water and 5% (B) 8% acetic acid in water, and C (Acetonitrile) was applied. The 10 µL of prepared sample extract was automatically injected, the flow rate was adjusted to 1 mL min^−1^, and separation was performed at 25 °C. Chromatograms were recorded at 280, 320, and 360 nm. The peaks of identified phenolic compounds were then quantified as mg kg^−1^ by comparing the results with a built-in library.

### 2.7. Sprouting of B. nigra for Biological Assessment

*B. nigra* was sprouted again on a larger scale under the same conditions for six days. The sprouts were gradually dried using the drying program for 24 h. The dried sprouts were then ground and extracted for biological evaluation [26]. Approximately 500 g of *B. nigra* sprouts was extracted with 2500 mL ethanol (50%) three times to prepare a *B. nigra* sprout hydroalcoholic extract (BNSE). A rotary evaporator was used to concentrate the filtered extract at 40 °C to evaporate the remaining solvent, and then it was frozen overnight and freeze-dried for 96 h at –52 °C and 0.032 mbar using CHRIST Alpha 1–2 LD plus (Osterode, Germany) [27]. The freeze-dried samples were pulverized using a porcelain morsel to prepare a homogeneous powder, and then they were kept in dark packages at 4 ± 1 °C until use.

### 2.8. Rat Experiment Design

Thirty-two male Wistar rats weighing between 180 and 200 g were used in this study. All experiments were approved by the Institutional Animal Ethics Committee (IAEC) at Qassim University, Saudi Arabia (Approval No. 23-19-08), issued Monday, 5 January 2023. The animals were housed in air-conditioned polypropylene cages under standard laboratory conditions at 24 ± 1 °C for one week for acclimatization. The rats were exposed to 12 h of light and 12 h of darkness and fed a commercial standard diet and water ad libitum. A cyclophosphamide (CYP) solution was prepared and intraperitoneally injected (i.p.) at 250 mg kg^−1^ as a single dose. The rats were then divided into four groups, with each group containing eight rats: G1: NR, normal rats; G2: CYP, injected a single dose of CYP at 250 mg kg^−1^ and did not receive any treatment; G3: CYP, injected a single dose of CYP at 250 mg kg^−1^ and administrated 250 mg Kg^−1^ of BNSE orally/daily; and G4: CYP, injected a single dose of CYP at 250 mg kg^−1^ and administrated 500 mg Kg^−1^ of BNSE orally/daily. At the end of the experiment, the rats were anesthetized, and blood samples were collected from the jugular vein. Immediately after the collection, the blood tubes were subjected to centrifugation at 4000 rpm at 10 °C for 30 min, and the obtained serum was preserved at −18 ± 1 °C until use.

### 2.9. Oxidative Stress Biomarkers

Reduced glutathione (GSH, µg dL^−1^) was estimated using a GSH colorimetric assay kit according to the method described by Beutler et al. [34]. Lipid peroxidation was estimated using a malondialdehyde (MDA, nmol mL^−1^) colorimetric assay kit by measuring thiobarbituric acid reactive substance (TBARS) and expressed in terms of MDA content according to Ohkawa et al. [35]. MDA, a product of fatty acid peroxidation, forms a colored complex upon reaction with thiobarbituric acid (TBA). The absorbance of the supernatant was measured at 532 nm, and the results were calculated as nmol mL^−1^. Superoxide dismutase (SOD, UL^−1^) activity using a SOD-type activity assay kit was determined according to Giannopolitis and Ries [36]. The color reaction was measured at 550 nm and is expressed as UL^−1^. Catalase (CAT, UL^−1^) activity was determined using a CAT activity assay kit according to the method of Aebi [37]. All oxidative stress biomarkers were determined using a blood chemistry analyzer (HumaLyzer 4000, HUMAN Gesellschaft für Biochemica und Diagnostica mbH, Wiesbaden, Germany).

### 2.10. Measurement of Hematological Parameters

Hematological parameters were determined as previously described [38] with minor modifications. Briefly, around five hundred microliters of blood from the tail vein of the rats was collected at the end of the 3rd week in a 1.5 mL^−1^ purple-capped tube incorporated disodium salt of ethylenediaminetetraacetic acid (EDTA-2Na). The numbers of white blood cells (WBCs), lymphocytes, and neutrophils were measured using a CBC colter (Mindray BC-2800 vet, Shenzhen Mindary Bio-Medical Electronics Co., LTD, Shenzhen, China); the results are presented as obtained.

### 2.11. Immunoglobulin, Pro-Inflammation, and Anti-Inflammation Cytokine Assay

Quantitative and qualitative analyses of leukocytes were carried out according to the descriptive protocol of Khan et al. [39]. The quantitative analysis of IgG (ab189578, Rat IgG ELISA Kit, Japan), IgA (ab157735, Rat IgA ELISA Kit, Abcam Co. Waltham, MA, USA), and IgM (ab157738, Rat IgM ELISA Kit, Abcam Co. Waltham, MA, USA) was conducted using ELISA-based techniques according to the kit instructions and Juto and Settergren [40]. Some cytokines for determining pro-inflammation, such as IL1-β (ab255730, Rat IL-beta ELISA Kit, Abcam Co. Waltham, MA, USA) and TNF-α (ab236712, Rat TNF-α ELISA Kit, Abcam Co. Waltham, MA, USA), and anti-inflammation, such as IL10 (ab214566, Rat IL10 ELISA Kit, Abcam Co. Waltham, MA, USA), IL-13 (ab269547, Rat IL-13 ELISA Kit, Abcam Co. Waltham, MA, USA), and IL-6 (ab234570, Rat IL-6 ELISA Kit, Abcam Co. Waltham, MA, USA), were examined according to Brink et al. [41].

### 2.12. Statistical Analysis

A statistical analysis was performed using SPSS (Ver. 22.0 for Windows, IBM, Houston, TX, USA). The experimental results are expressed as mean ± SE. Statistical significance was tested with a one-way ANOVA followed by a post hoc test, and *p*-values < 0.05 were applied according to Steel [42].

## 3. Results and Discussion

### 3.1. Total Phenolics and Antioxidant Capacities of B. nigra Seeds and Their Sprouts

Figure S1 shows the sprouting development of the BN seeds up to 7 days at 17 ± 1 °C and ~94% RH. It is obvious that the BN seeds had a high sprouting power, grew very quickly, and had well-developed stems and leaves, giving a high seeding rate and yield, which could be doubled in dry weight after 7 days. Phytochemicals such as TPC and related antioxidant activity (AOA) were assessed using DPPH and ABTS radical scavenging assays in the BN seeds and the BN sprouts for up to 7 days, and the data are illustrated in Table 1. The TPC in raw *B. nigra* seeds was about 37.91 mg GAE g^−1^ seeds dw. Increases in the TPC concentration were seen as the sprouting process progressed. No significant difference was recorded in TPC between the 6th and 7th days. When comparing the results of the BN seeds and the BN sprouts on the 6th day, a 2.8-fold increase in the TPC content was recorded. The results in the same table indicate significant increases in the AOA of the BN seeds and the BN sprouts up to 7 days at 17 ± 1 °C and ~94% RH. The AOA of the BN seeds to the BN sprouts assessed using DPPH and ABTS radical scavenging assays increased with the increasing TPC. The raw BN seeds indicated 77.52 and 118.05 µmol TE g^−1^ for DPPH and ABTS radical scavenging activities, respectively. A gradual increase in the AOA of the BN sprouts was observed, reaching 152.38 and 213.52 µmol TE g^−1^ after six days of sprouting for DPPH and ABTS radical scavenging activities, respectively. The increasing %s were 96% and 81% for AOA measured using BPPH and ABTS radical scavenging methods, respectively.

The amount of antioxidants and phenolic compounds increased during the sprouting process [43]. Unfortunately, the initial TPC and associated AOA were marginally reduced during the sprouting phase due to the washing and soaking processes. Some bioactive substances may leach out when osmotic pressure forces their immigration. Because of this, the TPC and associated AOA were impacted [44]. TPCs cause more AOAs to be produced, which has antioxidant and ameliorative benefits [45,46,47]. However, throughout the sprouting process, additional flavonoids and flavanols were synthesized [48]. These results are consistent with those of Efrem et al. [49], who indicated that the TPC of mustard seed after 1, 2, and 3 days increased by 16, 17, and 24%, respectively. Thus, sprouting is a better technique to enhance the nutritional and therapeutical potential of mustard seeds and can be used to develop such functional foods. Indeed, the polyphenols in plants are considered by-products of metabolism and are thought to provide health benefits associated with reducing the risk of chronic diseases [50]. Phenolic compounds are a type of antioxidant, and they have received a lot of praise for their capacity to scavenge free radicals linked to metabolic disorders [51,52]. Increased antioxidant activity can be achieved using phenolic compounds [53]. The antioxidant strength increases with increasing quantities of phenolic compounds [45,54,55,56]. However, the current work intends to conduct a basic investigation into the bio-changes in and fate of the polyphenols in BN sprouts. It suggests that the sprouts of BN seeds may be a unique source of active compounds with superior antioxidants [57]. Also, this could be due to the abilities of newly generated antioxidants to combat free radicals and reduce the oxidative stress that prevents degenerative diseases, and they may boost immunity [58].

### 3.2. Total Flavonoid and Flavonol Content in B. nigra Seeds and Their Sprouts

The results in Table 2 illustrate the TF and TFL content in the BN seeds and the BN sprouts up to 7 days at 17 ± 1 °C and ~94% RH. The TFs and TFLs were 19.34 and 6.34 mg QE g^−1^ in the BN seeds, respectively. A significant decrease in the TF content was observed after one day, while no significant decrease in the TFLs was noted. Interestingly, the TF and TFL contents increased with the progression of the sprouting period. Up to the 6th day, the TFs and TFLs reached their highest levels. Consequently, no significant differences were observed in the TF and TFL content between the 6th and the 7th days. Our results are similar to those obtained by Efrem et al. [49], who found that the TF content was 20.92 and 34.81 mg rutin equivalent g^−1^ in *B. nigra*. In comparison, it was lower than that noted by Li et al. [59], who found 14.92 mg g^−1^. Notably, the amount of phenolics and their derivatives, as well as antioxidants, rose during sprouting [43] and at the start of sprouting, whereas washing and soaking lowered the amount of phenolics due to osmotic pressure [44]. As confirmed, the newly created phenolics increased the TF contents and improved antioxidative and ameliorative efficiency [45,46,47]. Meanwhile, sprouting produced additional flavonoids and flavanols [48]. However, it is important to note that many agricultural, environmental, handling, and laboratory factors can significantly impact the number and content of phenolics in seeds [60].

### 3.3. Total Carotenoid Content in B. nigra Seeds and Their Sprouts

Table 3 illustrates the TC content in the BN seeds and BN sprouts up to 7 days at 17 ± 1 °C and ~94% RH. The seeds of *B. nigra* demonstrated 3.93 mg g^−1^. On the first day, the *B. nigra* seeds sprouted and showed substantially different results from the raw seeds, indicating a significant increase in TCs. The TC content increased significantly with the progression of sprouting time. These findings correlated with increased TPC, AOA, TF, and TFL content and could be effectively explained by the high AOA of the BN sprouts. Our results are higher than those obtained by Li et al. [59], who indicated that TC was 0.64 mg g^−1^ in BN seeds and found an increasing rate of photosynthetic pigment accumulation during sprouting. The important factors affecting TC concentrations include plant species, growing conditions, harvest ripeness, post-harvest storage, and extraction methods [60].

### 3.4. Identification and Quantification of Phenolic Compounds in B. nigra Seeds and Their Sprouts

Table 4 and Figure S2 illustrate the individual phenolic acids and flavonoids in the BN seeds and BN sprouts up to 7 days at 17 ± 1 °C and ~94% RH. Extracts from the BN seed sprouts on the 3rd, 6th, and 7th days were quantitatively analyzed for individual phenolic acids and flavonoids; the results are shown in Table 4. The BN seeds presented ten distinct phenolic acids and four different flavonoids. The highest phenolic acid concentration was 286.56 mg Kg^−1^ for syringic acid, followed by 127.70 mg Kg^−1^ for benzoic acid and 105.22 mg Kg^−1^ for *p*-Hydroxy benzoic acid. For the 3rd day sprouts, 16 phenolic acids and 7 flavonoids were quantified. Syringic acid (499.82 mg Kg^−1^) was the most abundant phenolic, followed by rosmarinic acid (403.72 mg Kg^−1^), benzoic acid (274.47 mg Kg^−1^), and *p*-Hydroxy benzoic acid (251.30 mg Kg^−1^). On the 6th day, the sprouts exhibited 13 phenolic acids and 6 different flavonoids, predominantly with benzoic acid and quercetin, respectively. While on the 7th day, the sprouts exhibited nine phenolic acids and six flavonoids, predominantly with rosmarinic acid and kaempferol, respectively.

On the 6th day, phenolics increased significantly, like caffeic acid, chlorogenic acid, cinnamic acid, ferulic acid, *O*-coumaric acid, benzoic acid, and rosmarinic acid. However, phenolics, such as catechol, *p*-Hydroxy benzoic acid, ellagic acid, *p*-coumaric acid, and syringic acid, decreased. Also, flavonoids, such as kaempferol and myricetin, increased, but catechin, quercetin, rutin, and resveratrol decreased significantly. When calculating the total phenolic acids and flavonoids, it was noted that the highest content was observed on the 6th day. Regarding the flavonoid content, both the BN seeds and BN sprouts were rich in flavonoid content. In the BN seeds after 3 days, quercetin presented the highest content among the quantified flavonoids, while myricetin presented the highest content after 6 days. In contrast, kaempferol presented the highest content among the quantified flavonoids on the 7th day.

The obtained results are in agreement with those obtained by Lee et al. [61], who identified and quantified valuable contents of different phenolics, such as gallic acid, catechin, epicatechin, myricetin, quercetin, and rutin, in different extracts of *B. nigra*. HPTLC was used to conduct a further compound analysis to quantify phenolics and identify the chemical constituents responsible for their antioxidant activity. The HPTLC analysis quantified the major phenolic compounds in the BN extract. It quantified several compounds, such as gallic acid, quercetin, ferulic acid, caffeic acid, and rutin, with gallic acid being predominant [17]. Recently, Nicácio et al. [62] evaluated the QuEChERS method for determining phenolics in *B. alba*, *B. juncea*, and *B. nigra* seeds, and their results highly agree with ours. They identified and quantified valuable contents of 4-hydroxybenzoic acid, vanillic acid, caffeic acid, syringic acid, chlorogenic acid, *p*-coumaric acid, ferulic acid, sinapic acid, gallic acid, ellagic acid, apigenin, luteolin, procatechuic acid, (+)-catechin, (-)-epicatechin, (-)-epicatechin gallate, chrysin, kaempferol, quercetin, myricetin, and naringenin in *B. nigra*.

### 3.5. Effects of B. nigra Sprout Extract on Antioxidant Biomarkers in CYT-Induced Immunosuppression in Rats

As shown in Table 5, the injection of the immunosuppressant and oxidative stress agent CYP dramatically decreased GSH, CAT, and SOD enzyme levels. However, the serum MDA levels of the CYP-treated rats were elevated compared to those of the NR group. Remarkable increases in the activity of the antioxidant enzymes GSH, CAT, and SOD, as well as significant decreases in MDA levels, were seen in the rats treated with an extract of *B. nigra* sprouts (BNS), as shown in Table 5. For GSH, the enhancement rates for the BN extract at 250 and 500 mg kg^−1^ were 49.55 and 78.69%, respectively. Similarly, the antioxidant capacity in rats was accelerated to 30.42 and 48.47% for CAT and 40.50 and 81.35% for SOD in the BN sprouts at 250 and 500 mg kg^−1^, respectively. High levels of antioxidants helped fight the autoxidation process, resulting in MDA reductions of 23.72 and 50.37% in the BN sprouts at 250 and 500 mg kg^−1^, respectively. According to the findings, doses of the BN extract at 250 and 500 mg kg^−1^ significantly elevated glutathione (GSH), catalase (CAT), and superoxide dismutase (SOD) and reduced malondialdehyde (MDA) levels. These observations indicate that BN sprouts at 250 and 500 mg kg^−1^ exhibit superior antioxidant activity in immunosuppressed rats.

It is known that CYP affects antioxidant enzymes, significantly increasing oxidative stress, as Golmohammadi et al. [63] confirmed. Our result agrees with that of Rajamurugan et al. [18], who indicated that a crude methanolic extract of *B. nigra* exhibits antioxidative stress, accelerates the production of oxidative enzymes, and scavenges the free radicals in biological systems. This may be due to its superior content of phytochemicals and bioactive substances [11,64]. It is widely known that oxidative damage induced by CYP hepatotoxicity can be prevented by administering plant extracts with antioxidant activity [63]. The results of the current investigation corroborate this finding and show that the BN extract raised the levels of GSH, CAT, and SOD. Our results agree with those of Keshari et al. [65] since it was found that the stress-induced production of antioxidant defense enzymes and intracellular GSH modification required the activation of the Nrf2/ARE pathway [66]. Indeed, one of the strategies by which functional foods have been shown to benefit disease prevention is through their antioxidant capacity [45,67], controlling blood pressure, communicating with the gut microbiome, dampening the overproduction of pro-inflammatory cytokines, and stimulating antioxidant enzymes.

### 3.6. Effects of B. nigra Sprout Extracts on Hematological Markers in CYP-Induced Immunosuppression in Rats

Table 6 shows the WBCs, lymphocytes, and neutrophils involved in CYP-induced immunosuppression in rats. Rats with low WBC counts have compromised immune systems. Compared to the NR group, CYP significantly decreased the WBCs, lymphocytes, and neutrophils in the blood, indicating severe immune suppression. The WBCs, lymphocytes, and neutrophils decreased dramatically after administering the BN extract, as shown in Table 6.

Interestingly, the BN extract at 250 and 500 mg kg^-1^ (*p* > 0.05) showed substantial improvements, indicating that both dosages mitigated the CYP-induced immune suppression in a dose-dependent manner. The best results were seen with the BN extract at 500 mg kg^−1^, which was even better than the BN extract at 250 mg kg^−1^. Similarly, the ethanolic extract of garlic demonstrated immunomodulatory and antioxidant activities and could diminish the alterations induced by CYP [68]. However, WBCs, as an indicator of immune system health, were increased after consuming either raw or fermented turmeric camel milk, both beneficial in enhancing immunity and shielding against oxidative stress [64,69].

### 3.7. Effects of B. nigra Sprout Extract Administration on Immunoglobulins in CYP-Induced Immunosuppression in Rats

Biomarkers of humoral immunity, such as immunoglobulins (Igs), are extremely important. Three Ig types, namely, IgG, IgM, and IgA, account for nearly all serum Igs [70]. The serum concentrations of IgA, IgG, and IgM were determined using appropriate ELISA kits to examine the impact of the BN sprout extract on humoral immunity in the immunosuppressed rats. Table 7 shows that CYP inhibited humoral immune function because the IgG, IgM, and IgA concentrations were lower in the CPY group than in the NR group (*p* < 0.05). In contrast, there was a statistically significant rise (*p* < 0.05) in the IgG, IgA, and IgM concentrations in the CYP+BN 250 mg kg^−1^ and CYP+BN 500 mg kg^−1^ groups. However, the BN extract at 250 and 500 mg kg^−1^ recovered Ig production by 27.51 and 68.78% for IgG, 49.29 and 68.57% for IgA, and 110 and 173% for IgM, respectively. In a dose-dependent way, BN extract administration improved humoral immunity in the CYP-immunosuppressed rats; several Igs were even more accelerated than in the NR group.

Numerous plant-based compounds with immune-modulating properties have been approved [71,72,73,74,75]. There is evidence that IgG antibodies can bind to the FC receptor on NK and macrophage cells, altering the activities of these immune system cells [76]. IgA may increase pathogen adherence to mucosal cells, making it equally effective against viruses and bacteria [77]. When combined with complement and macrophages, IgM can stimulate complement and improve phagocytosis [78]. Regarding the immune system, T cells are associated with the cell-mediated response, whereas B cells are linked to the humoral response [79]. Antigen-stimulated T cells undergo a process of differentiation and transformation into plasma blasts, which release immunoglobulin and control immunological responses [80]. The substantial increase in plasma IgA, IgG, and IgM levels was caused by the BN extract in the CYP-injected rats, suggesting that the BN extract can improve the humoral immune response in immunosuppressed rats. Different plant extracts and spices have shown the same results [81,82]. Han et al. [83] stated that barley leaf extract effectively improves immunological manifestations by stimulating the development of Th1 and Th2 cells through the JAK/STAT1/T-bet signaling and TLR2/GATA3 signaling pathways, respectively. The oral administration of barley leaf extract significantly protects CYP-treated immunosuppressed rats’ weakened immune system by recovering body weight, splenocyte proliferation, and NK cell cytotoxic activity. The high content of flavonoids present in our BN extract, as shown in Table 4, supports the findings of Ebokaiwe et al. [84], as they revealed that quercetin ameliorates the effect of CYP-instigated IDO/TDO activities in the cerebral cortex and hippocampus by restoring antioxidant enzymes and preventing oxidative-inflammatory stress.

### 3.8. Effects of B. nigra Sprout Extracts on Pro- and Anti-Inflammatory Cytokines in CYP-Induced Immunosuppression in Rats

Cytokines are signaling proteins that regulate T helper cell activities [85]. Compared to the NR group, the CYP group had considerably reduced amounts of IL-1, IL-6, IL-10, IL-13, and TNF-α, as indicated in Table 8. There was a notable increase in the production of identified cytokines compared to in the CYP group (*p* < 0.05). Interestingly, in the CYP-immunosuppressed rats, the BN extract at both dosages significantly increased Th1 and Th2 cytokine production [83]. The extract of BN considerably amplified immunological activity in the immunosuppressed rats, with the augmentation of cytokine production being the most pronounced at 500 mg kg^−1^ compared to at 250 mg kg^−1^ [66]. Interleukins regulate NK cells’ cytotoxic actions and stimulate B lymphocyte growth and differentiation [86]. Leukocytes and blood vessel endothelial cells regulate the early inflammatory responses via the inflammatory cytokines IL-1β, IL-6, and TNF-α. IL-1β is produced uniquely by activated monocytes and macrophages and cooperates with several immune cells, including endothelial cells [87]. TNF-α is a key pro-inflammatory cytokine that results in the production of IL-6 and other cytokines. IL-6 has many impacts, including T-cell activation and thymocyte and B-cell differentiation [88]. In general, Th1 cytokines promote lymphocyte division and proliferation, DC maturation, and Th1-based immune responses [89]. Th2 cytokines, including IL-6, IL-10, and IL-13, enhance Th2-type immune responses and antibody production [90]. Thus, the BN extract potentiated immunological activity in the immunosuppressed rats.

## 4. Conclusions

During the primary sprouting experiment, *B. nigra* sprouts were evaluated by following the bio-change in their bioactive compounds. Consequently, phenolic acids, their derivatives, and flavonoids were identified and quantified using HPLC. Additionally, in CYP-induced immunosuppression in rats, the antioxidant and immune-boosting characteristics of the hydroalcoholic extract of *B. nigra* sprouts were examined for the first time. BN extracts at 250 and 500 mg kg^−1^ were orally administrated to the CYP-immunosuppressed rats for 3 weeks. The BN sprouts exhibited incredible TPC, TFs, TFLs, TCs, and AOA. Individual phenolic acids and flavonoids were increased in number and quantity as newly synthesized compounds during sprouting up to the 6th day. An in vivo analysis in the rat model indicated that doses of 250 and 500 mg kg^−1^ significantly improved antioxidative stress and the number of WBCs, lymphocytes, and neutrophils in the blood, indicating stimulation of the immune response by increasing Igs such as IgG, IgM, and IgA in a dose-dependent manner. Moreover, 500 mg kg^−1^ of the BN extract stimulated cytokine production more so than 250 mg kg^−1^, showing that the extract considerably boosted immunological activity. It could be observed that the *B. nigra* sprout extracts have immunosuppressive properties against CYP-induced immunosuppression in rats.

## Figures and Tables

**Table 1:** Total phenol content and antioxidant activity in *B. nigra* seeds and their sprouts at 17 ± 1 °C and ~94% RH, (mean ± SE), *n* = 6.

Sprouting Time (Day)	TPC (mg GAE g^−1^ dw)	DPPH (µmol TE g^−1^ dw)	ABTS (µmol TE g^−1^ dw)
Raw seeds	37.91 ± 2.31 ^f^	77.52 ± 5.13 ^d^	118.05 ± 6.14 ^c^
1	55.44 ± 4.11 ^e^	68.36 ± 8.02 ^e^	96.66 ± 8.27 ^c^
2	53.84 ± 2.03 ^e^	78.45 ± 2.44 ^d^	97.09 ± 11.69 ^c^
3	74.85 ± 2.64 ^d^	109.09 ± 5.88 ^c^	159.49 ± 19.17 ^b^
4	84.22 ± 2.44 ^c^	114.92 ± 4.23 ^c^	169.8 ± 11.31 ^b^
5	92.70 ± 2.23 ^b^	133.58 ± 6.17 ^b^	187.75 ± 12.93 ^ab^
6	104.58 ± 3.25 ^a^	152.38 ± 8.48 ^a^	213.52 ± 14.32 ^a^
7	100.91 ± 3.11 ^a^	150.54 ± 3.08 ^a^	209.39 ± 1.17 ^a^

TPC: total phenolic content, DPPH-RSA: 2,2-diphenyl-1-picrylhydrazyl radical scavenging activity, ABTS: 2,2′-azino-bis(3-ethylbenzothiazoline-6-sulfonic acid) radical scavenging activity, ^a,b,c,d,e,f^: no significant difference (*p* > 0.05) between any two means within the same column that have the same superscript letter.

**Table 2:** Total flavonoid and flavonol contents in *B. nigra* seeds and their sprouts at 17 ± 1 °C and ~94% RH, (mean ± SE), *n* = 6.

Sprouting Time (Day)	TF (mg QE g^−1^ dw)	TFL (mg QE g^−1^ dw)
Raw seeds	19.34 ± 1.26 ^de^	6.34 ± 0.20 ^g^
1	6.56 ± 1.72 ^f^	4.90 ± 0.18 ^g^
2	17.33 ± 1.11 ^e^	11.8 ± 0.21 ^f^
3	28.09 ± 1.99 ^cd^	22.96 ± 0.41 ^e^
4	37.29 ± 4.69 ^c^	26.45 ± 1.40 ^d^
5	74.21 ± 5.58 ^b^	33.47 ± 1.41 ^c^
6	111.23 ± 4.46 ^a^	39.66 ± 1.56 ^a^
7	105.65 ± 2.78 ^a^	36.30 ± 0.64 ^b^

TF: total flavonoid content, TFLs: total flavonols, ^a,b,c,d,e,f,g^: no significant difference (*p* > 0.05) between any two means within the same column that have the same superscript letter.

**Table 3:** Total carotenoid content in *B. nigra* seeds and their sprouts at 17 ± 1 °C and ~94% RH (mean ± SE), *n* = 6.

Sprouting Time (Day)	Carotenoids (mg g^−1^ dw)
Raw seeds	3.93 ± 0.36 ^c^
1	7.76 ± 0.97 ^b^
2	8.10 ± 0.31 ^b^
3	8.25 ± 0.85 ^b^
4	8.71 ± 0.66 ^b^
5	9.52 ± 1.56 ^ab^
6	11.63 ± 1.05 ^a^
7	11.75 ± 0.61 ^a^

^a,b,c^: no significant difference (*p* > 0.05) exists between any two means within the same column with the same superscript letter.

**Table 4:** Quantitative analysis of phenolic compounds in *B. nigra* seeds and their sprouts at 17 ± 1 °C and ~94% RH by HPLC-DAD.

Components	No.	Compound	mg kg^−1^			
			Raw	3 Days	6 Days	7 Days
Phenolics *	1	Pyrogallol	-	19.00	-	-
	2	Quinol	-	45.76	-	-
	3	3-Hydroxytyrosol	-	-	-	-
	4	Catechol	69.72	173.71	27.14	-
	5	*p*-Hydroxy benzoic acid	105.22	251.30	33.72	-
	6	Caffeic acid	41.52	47.68	237.97	-
	7	Chlorogenic acid	-	9.01	90.20	-
	8	Cinnamic acid	4.68	4.01	302.40	25.78
	9	Ellagic acid	-	21.44	9.26	-
	10	Vanillic acid	27.35	19.57	75.56	64.18
	11	Ferulic acid	11.46	27.52	28.25	7.16
	12	Gallic acid	90.72	88.55	-	4.05
	13	*O*-coumaric acid	-	3.55	12.55	16.92
	14	*p*-coumaric acid	10.71	25.28	16.87	11.16
	15	Benzoic acid	127.70	274.47	2182.20	129.23
	16	Rosmarinic acid	-	403.72	749.55	222.56
	17	Syringic acid	286.56	499.82	8.11	40.73
Flavonoids **	1	Catechin	333.44	154.59	25.13	511.82
	2	Kaempferol	-	58.62	636.49	5693.18
	3	Myricetin	57.08	128.29	3778.81	287.02
	4	Quercetin	1031.83	3013.09	2386.90	1782.54
	5	Rutin	-	162.37	111.06	41.31
	6	Resveratrol	464.90	693.76	583.31	129.25
	7	Naringenin	-	133.66	-	-

*: phenolic acids and derivatives of some of their acids (wavelengths of 280 and 320 nm); **: flavonoid wavelength (360 nm); -: not discovered in HPLC estimation.

**Table 5:** Effects of *B. nigra* sprout extract at different doses on antioxidant biomarkers in CYP-induced immunosuppression in rats (mean ± SE), *n* = 8.

Groups	Antioxidant Biomarkers			
	GSH (µg dL^−1^)	CAT (U L^−1^)	SOD (U L^−1^)	MDA (µ mol mL^−1^)
NR	93.78 ± 9.28 ^ab^	128.16 ± 8.17 ^a^	105.37 ± 5.97 ^b^	21.85 ± 1.99 ^c^
CYP	59.88 ± 5.35 ^c^	83.06 ± 7.34 ^d^	63.38 ± 3.17 ^d^	40.26 ± 4.11 ^a^
CYP+BN250	89.55 ± 5.79 ^b^	108.33 ± 8.59 ^c^	89.05 ± 2.89 ^b^	30.71 ± 3.09 ^b^
CYP+BN500	107.91 ± 7.64 ^a^	123.32 ± 9.72 ^a^	114.94 ± 4.59 ^a^	19.98 ± 1.49 ^c^

GSH: reduced glutathione, CAT: catalase, SOD: superoxide dismutase, MDA: malonaldehyde, ^a,b,c,d^: no significant difference (*p* > 0.05) between any two means within the same column with the same superscript letters.

**Table 6:** Effects of *B. nigra* sprout extract at different doses on WBCs, lymphocytes, and neutrophils in CYP-induced immunosuppression in rats (mean ± SE), *n* = 4.

Groups	Hematological Parameters		
	WBCs [10^9^ L^−1^]	Lymphocytes [10^9^ L^−1^]	Neutrophils [10^9^ L^−1^]
NR	9.72 ± 0.16 ^a^	8.38 ± 0.07 ^a^	1.19 ± 0.05 ^a^
CYP	6.29 ± 0.21 ^d^	5.11 ± 0.11 ^d^	1.03 ± 0.09 ^b^
CYP+BN250	8.12 ± 0.10 ^c^	6.91 ± 0.19 ^c^	0.89 ± 0.06 ^c^
CYP+BN500	9.24 ± 0.23 ^b^	7.69 ± 0.27 ^b^	1.31 ± 0.09 ^a^

WBCs: white blood cells, ^a,b,c,d^: no significant difference (*p* > 0.05) between any two means within the same column that have the same superscript letters.

**Table 7:** Effects of *B. nigra* sprout extract at different doses on immunoglobulins in CYP-induced immunosuppression in rats (mean ± SE), *n* = 8.

Groups	Immunoglobulins (mg mL^−1^)		
	IgG	IgA	IgM
NR	3.88 ± 0.58 ^a^	2.60 ± 0.18 ^a^	0.46 ± 1.80 ^a^
CYP	1.89 ± 0.73 ^b^	1.40 ± 0.05 ^c^	0.19 ± 0.08 ^b^
CYP+BN250	2.41 ± 0.75 ^c^	2.09 ± 0.17 ^b^	0.40 ± 0.02 ^a^
CYP+BN500	3.19 ± 0.63 ^d^	2.36 ± 0.23 ^ab^	0.52 ± 0.06 ^a^

^a,b,c^: no significant difference (*p* > 0.05) between any two means within the same column with the same superscript letters.

**Table 8:** Effects of *B. nigra* sprout extract at different doses on pro-inflammation and anti-inflammation cytokines in CYP-induced immunosuppression in rats (mean ± SE), *n* = 8.

Groups	Cytokines (ng mL^−1^)				
	IL-1 β	IL-6	IL-10	IL-13	TNF-α
NR	66.56 ± 5.23 ^a^	166.60 ± 3.04 ^b^	41.82 ± 4.62 ^a^	118.90 ± 2.73 ^a^	38.77 ± 3.46 ^a^
CYP	33.60 ± 4.78 ^c^	107.67 ± 5.67 ^c^	24.73 ± 3.22 ^c^	78.71 ± 0.71 ^d^	24.81 ± 2.06 ^b^
CYP+BN250	49.78 ± 4.32 ^b^	180.77 ± 7.72 ^b^	33.99 ± 3.34 ^b^	99.11 ± 2.22 ^c^	31.81 ± 3.93 ^ab^
CYP+BN500	69.7 ± 6.53 ^a^	207.40 ± 3.72 ^a^	44.34 ± 2.64 ^a^	110.38 ± 1.62 ^b^	37.02 ± 2.02 ^a^

^a,b,c^: no significant difference (*p* > 0.05) between any two means within the same row with the same superscript letters.

## Data Availability

Data are contained within the article.

## Supplementary Materials

The following supporting information can be downloaded at https://www.mdpi.com/article/10.3390/foods12193652/s1, Figure S1: The sprouting development of BN seeds over 7 days at 17 ± 1 °C and ~94% RH; Figure S2A: HPLC chromatogram of identified phenolic in *B. nigra* seeds; Figure S2B. HPLC chromatogram of identified phenolic in *B. nigra* sprouts on the 3rd day of sprouting at 17 ± 1 °C and ~94% RH; Figure S2C: HPLC chromatogram of identified phenolic in *B. nigra* sprouts on the 6th day of sprouting at 17 ± 1 °C and ~94% RH; Figure S2D: HPLC chromatogram of identified phenolic in *B. nigra* sprouts on the 7th day of sprouting at 17 ± 1 °C and ~94% RH.
