# Supplementary material for: Immune-Boosting Potentiating Properties of Brassica nigra Hydroalcoholic Extract in Cyclophosphamide-Induced Immunosuppression in Rats
Source: Foods. 2023 Oct 3;12(19):3652. doi: 10.3390/foods12193652 (PMC10572729; doi:10.3390/foods12193652)
Supplement: Supplementary file 1 [file foods-12-03652-s001.zip › foods-2576709-supplementary.pdf]

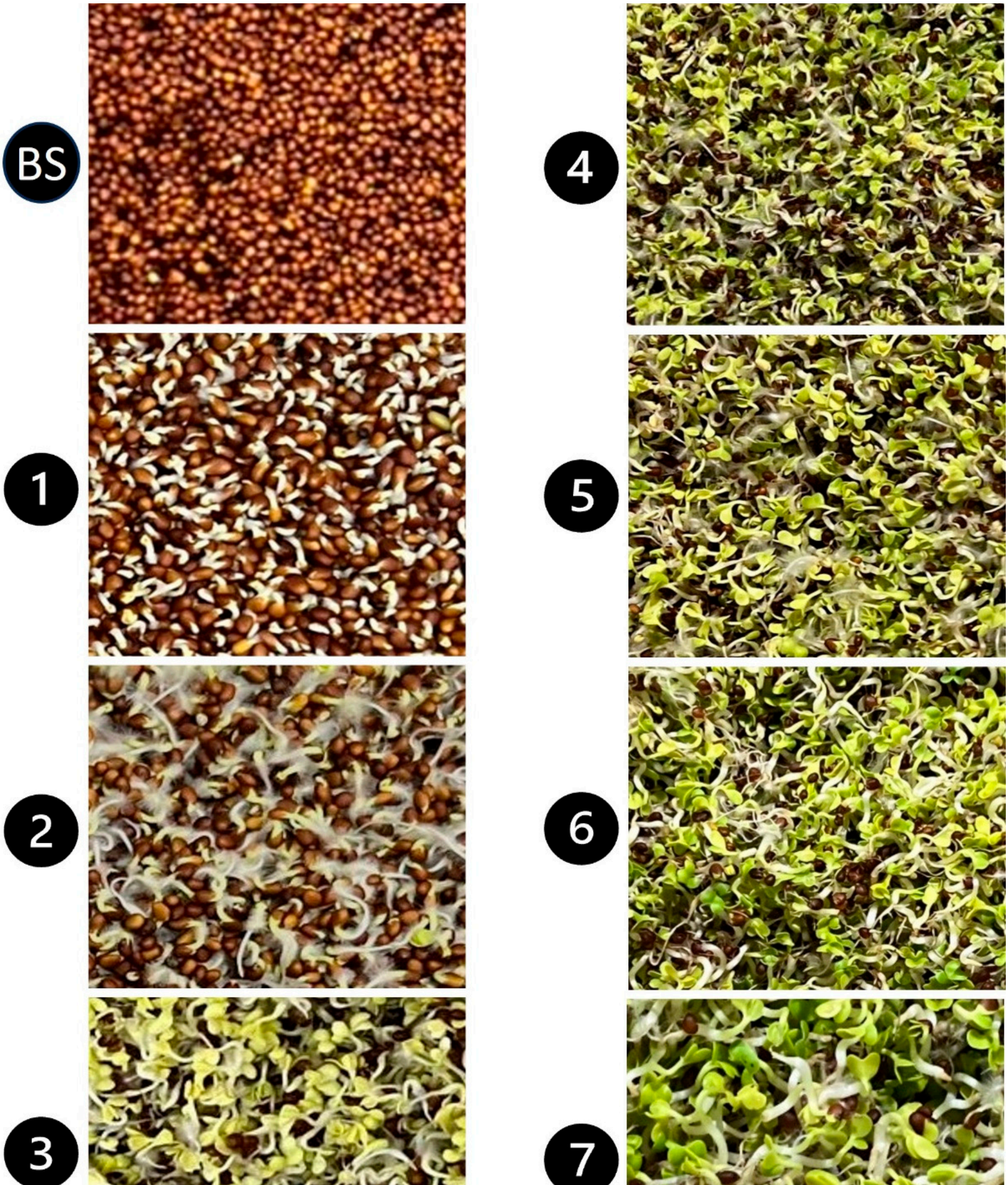

Figure S1. The sprouting development of BN seeds over 7 days at 17 ± 1 °C and ~94% RH.

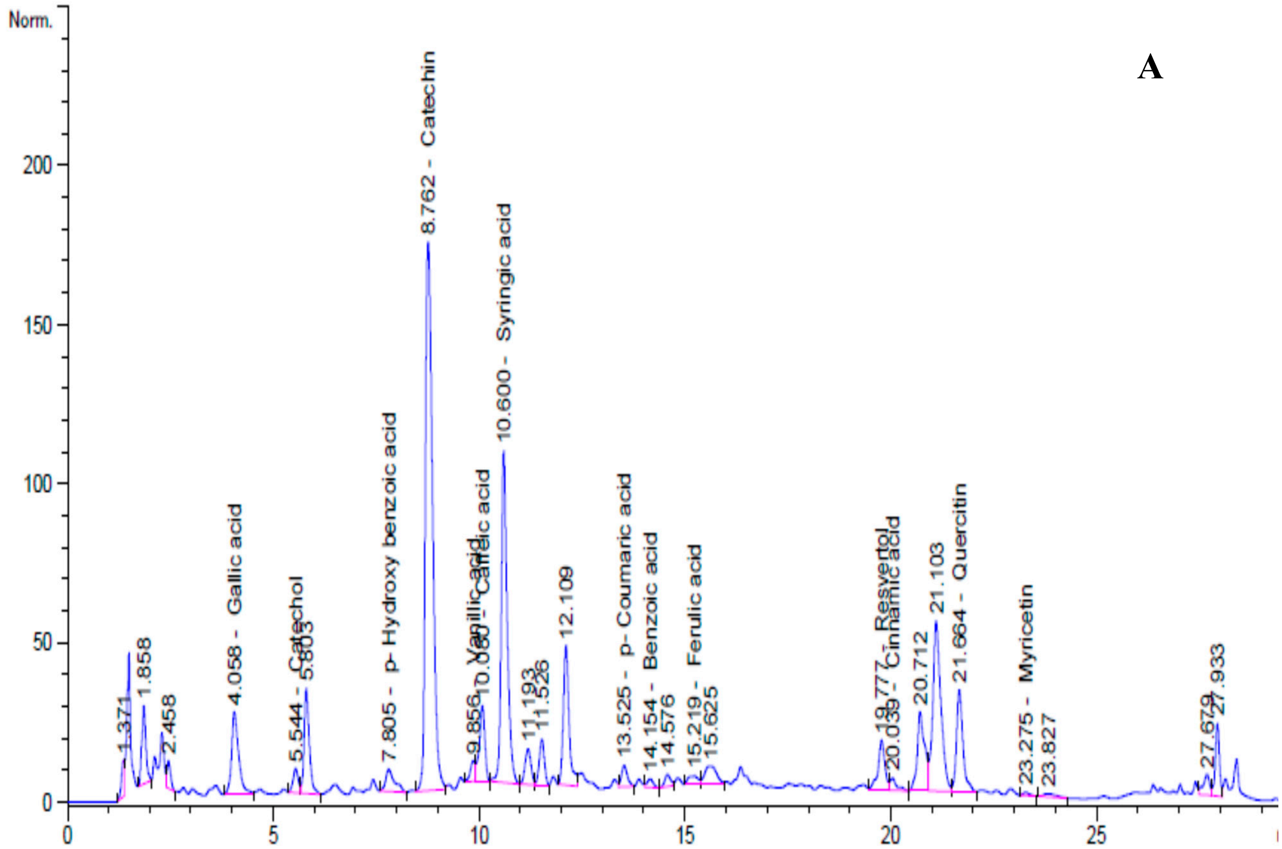

Figure S2A. HPLC chromatogram of identified phenolic in *B. nigra* seeds.

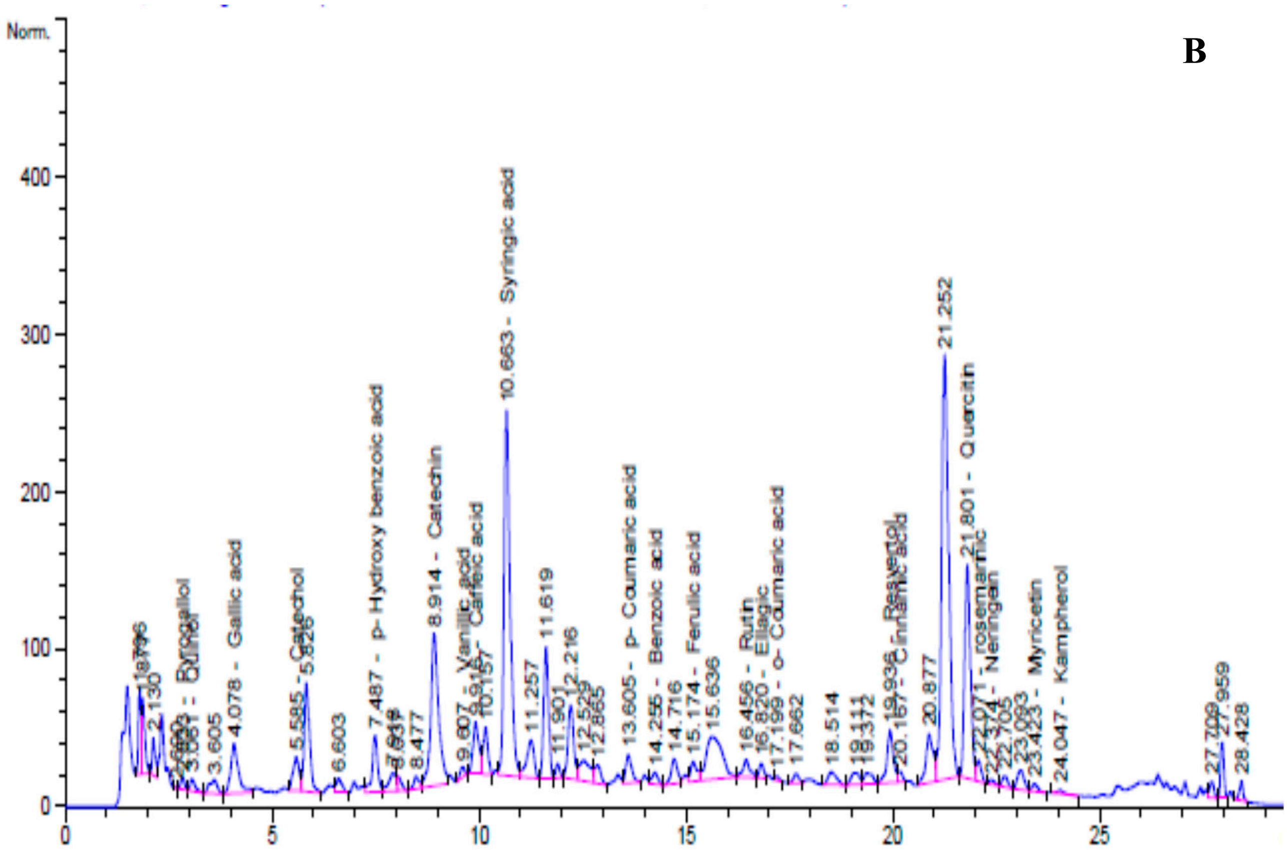

Figure S2B. HPLC chromatogram of identified phenolic in *B. nigra* sprouts on the 3rd day of sprouting at 17 ± 1 °C and ~94% RH.

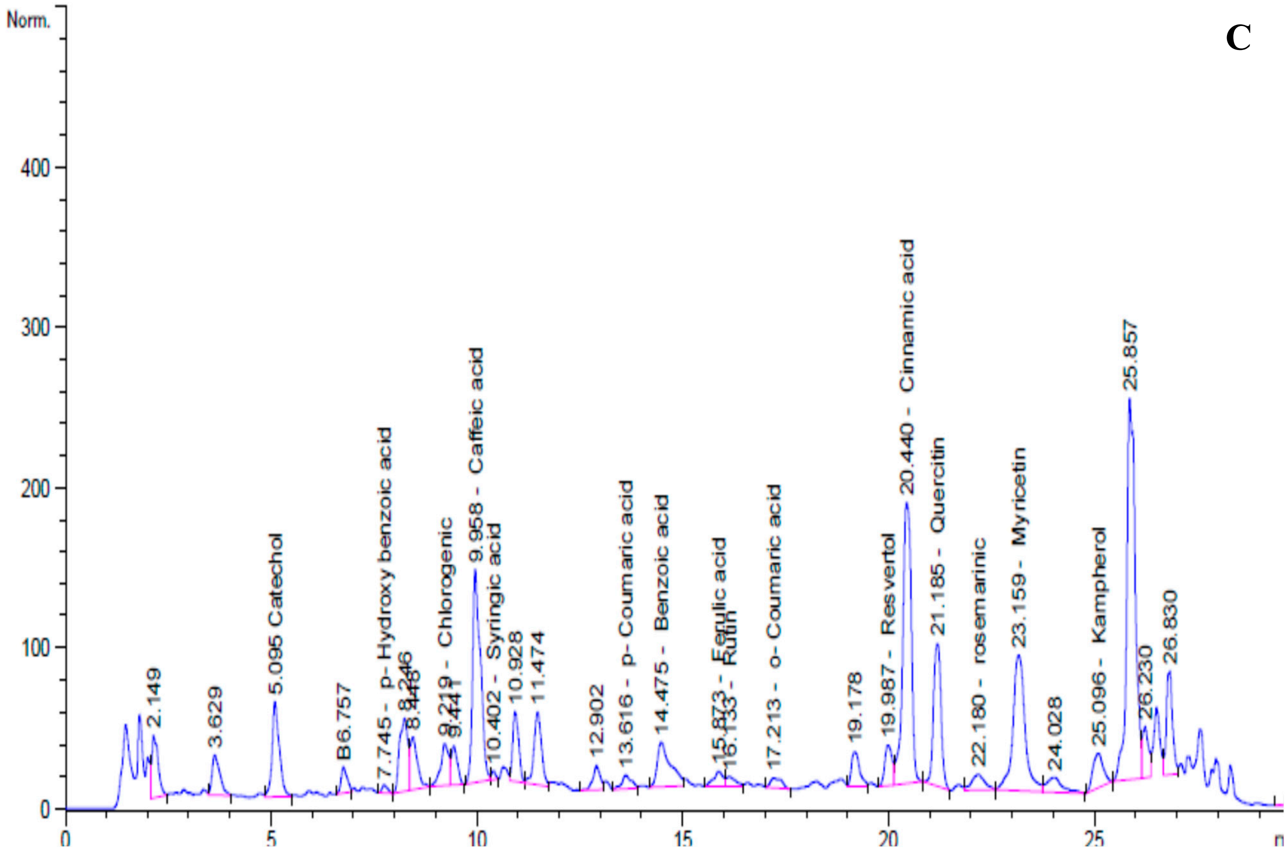

Figure S2C. HPLC chromatogram of identified phenolic in *B. nigra* sprouts on the 6th day of sprouting at 17±1°C and ~94% RH.

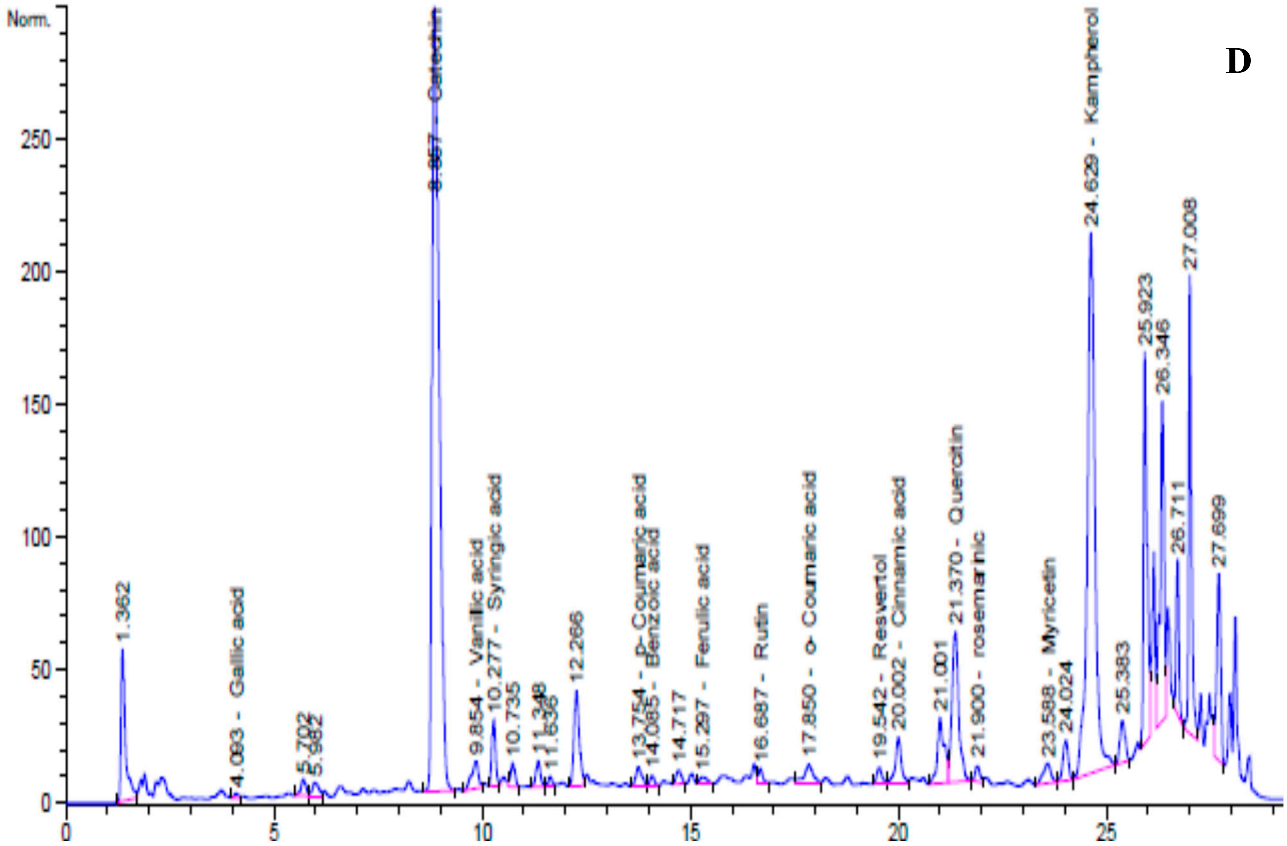

Figure S2D. HPLC chromatogram of identified phenolic in *B. nigra* sprouts on the 7th day of sprouting at 17 ± 1 °C and ~94% RH.
